# Enhancing Inactivated Yellow Fever 17D Vaccine-Induced Immune Responses in Balb/C Mice Using Alum/CpG

**DOI:** 10.3390/vaccines11121744

**Published:** 2023-11-22

**Authors:** Yadan Zhang, Rong Yang, Guangying Yuan, Weidong Li, Zihao Cui, Zhuangzhuang Xiao, Xiaofei Dong, Hongqiang Yang, Xiaojuan Liu, Le Zhang, Yirong Hou, Manyu Liu, Sushi Liu, Yu Hao, Yuntao Zhang, Xiaotong Zheng

**Affiliations:** Beijing Institute of Biological Products Company Limited, Beijing 100170, China; zhangyadan@sinopharm.com (Y.Z.); yangrong31@sinopharm.com (R.Y.); yuanguangying@sinopharm.com (G.Y.); liweidong21@sinopharm.com (W.L.); taurus_crab@126.com (Z.C.); xiaozhuangzhuang1@sinopharm.com (Z.X.); dongxiaofei@sinopharm.com (X.D.); yanghongqiang1@sinopharm.com (H.Y.); llxjj1236@163.com (X.L.); zhang1le23@163.com (L.Z.); hyr18822084262@163.com (Y.H.); manyuliufirst2017@126.com (M.L.); sushiliu0125@163.com (S.L.); supeihao1@sina.com (Y.H.)

**Keywords:** adjuvant, CpG, yellow fever, vaccine, immunogenicity

## Abstract

There are some concerns about the safety of live attenuated yellow fever vaccines (YF–live), particularly viscerotropic adverse events, which have a high mortality rate. The cellular production of the vaccine will not cause these adverse effects and has the potential to extend applicability to those who have allergic reactions, immunosuppression, and age. In this study, inactivated yellow fever (YF) was prepared and adsorbed with Alum/CpG. The cellular and humoral immunities were investigated in a mouse model. The results showed that Alum/CpG (20 μg/mL) could significantly increase the binding and neutralizing activities of the antibodies against YF. Moreover, the antibody level at day 28 after one dose was similar to that of the attenuated vaccine, but significantly higher after two doses. At the same time, Alum/CpG significantly increased the levels of IFN-γ and IL-4 cytokines.

## 1. Introduction

YF is a mosquito-borne viral illness that has caused a large number of diseases and deaths for centuries [1]. YF outbreaks continue to occur, resulting in an estimated 78,000 annual deaths, without considering under-reporting [2]. At present, YF remains a major public health threat to millions of individuals in tropical South America and many locations in Sub-Saharan Africa. However, there is a continuous risk of YF being introduced into new areas due to international travel to and from at-risk areas [1]. The drivers of YF virus (YFV) circulation, such as climate, vector factors, and population movement, make YF a huge threat to populations around the world. As an infection transmitted via the bite of the infected Aedes species of mosquito, YF should be controlled via vectors [3]. In fact, vector control approaches are usually holistic in areas where YF is endemic, striving to include chemical, biological, and environmental methods [4]. However, most control programs are challenged by one or multiple variables, such as environmental change, insecticide resistance, population growth, urbanization, and climate change. And, vector control has failed to arrest the expanding geographic distribution of arboviruses. Additionally, no specific anti-viral treatment has been approved for YFV infections [5]. The case fatality rate has been estimated as 50% in hospitalized patients infected with YF [6]. Therefore, the vaccine is still one of the most effective methods to control YF.

YF is caused by an arbovirus of the family Flaviviridae, which is a positive-sense, single-stranded RNA ([+]ssRNA) virus. Mature virions of 50 nm diameter are icosahedral and comprise a nucleocapsid composed of capsid (C) protein subunits. The viral envelope is studded with dimers of the envelope (E) glycoprotein and membrane (M) protein [7]. Currently, YF is prevented by the live attenuated vaccine, termed 17D, which was formulated after numerous passages of the wild-type Asibi strain in embryonated chicken eggs [2]. However, the live attenuated vaccine might lead to YF vaccine-associated neurological disease (YEL-AND) and YF vaccine-associated viscerotropic disease (YEL-AVD), which are caused by the live attenuated virus attacking the central nervous system and visceral organs, respectively. These serious adverse events are rare, but potentially fatal [8,9]. Moreover, egg-based live attenuated vaccines can cause allergic reactions, but cannot be produced in large quantities [10,11]. The traditional manufacturing practices based on the propagation of attenuated YFV in chicken embryos can only generate from 300 to 400 doses per egg. Annual vaccine production by all of the manufacturers in the United States, France, Senegal, the People’s Republic of China, the Russian Federation, and Brazil, which are the only six countries which have the ability to produce YF vaccines, totals approximately 80 million doses. This yield cannot meet either the ‘vaccine needs’ to eliminate YF epidemics from 2017 to 2026, about 138.4 million doses per year, or the doses required for a pandemic [8]. During the outbreak of YFV in Brazil, the emergency use of the fractional dose had to be introduced to increase the vaccination rates, despite Brazil being a major YF vaccine producer [10]. At the same time, the YF vaccine, currently on the market, is not available to immunosuppressed or thymectomized patients, children under 9 months of age, people with egg-protein allergies, and lactating women. In addition, inactivated vaccines need to be stored at 4 °C, while live attenuated vaccines need to be stored in ultra-cold conditions, which makes the former more convenient. In view of these problems, there is a growing need to develop new YF vaccine candidates. The inactivated vaccine based on a Vero cell culture would overcome the problems of YEL-AND, YEL-AVD allergic reactions [8]. In addition, A 320-L bioreactor batch would result in about 1 million doses at full capacity. To produce the same amount of egg-based vaccine doses, about 2500–3500 SPF eggs are required [10]. The bioreactor could increase annual global vaccine production capacity. Compared with the live attenuated vaccine, the use of an adjuvant would likely be required to induce a protective immune response to the inactivated vaccine.

Aluminum is one of the most widely-used adjuvants, which is extensively used for the development of most types of vaccine [12]. Aluminum-based adjuvants are safe and could induce a strong humoral response, but they are not good at eliciting a cellular immune response [12]. Therefore, aluminum is perceived as a relatively ‘weak’ vaccine adjuvant [13]. XRX-001 is an investigational YF inactivated vaccine manufactured by Xcellerex from the 17D strain grown in Vero cells, and the virus is adsorbed to 0.2% aluminum hydroxide (alum) [14]. Unlike the YF live attenuated vaccine, which could provide life-long protection for 99% of vaccinated people with a single dose [10], two injections of the YF inactivated vaccine are required for effective immunization [11,14]. This phenomenon may be related to the inactivation of the virus, and aluminum is not sufficient to compensate for the deficiency [13]. The addition of a new adjuvant with a significant cellular immune induction ability may make up the deficiency of the aluminum adjuvant and effectively improve the immunogenicity of the vaccine [13,15].

CpG ODN is an oligodeoxynucleotide containing an immunostimulatory sequence that directly activates human B cells and plasmacytoid dendritic cells via Toll-like receptors to generate greater cellular responses, enhancing the immune response [16,17]. The approval of the hepatitis B vaccine (HepB-CpG) from the Food and Drug Administration (FDA) in 2017 enabled CpG application to human vaccines for the first time [18]. Previous studies have suggested that the immunostimulatory properties of CpG 7909 makes the hepatitis B vaccine generate a robust and durable immune response [19]. The addition of CpG 7909 to BioThrax enhanced the immune responses and shorten the course of immunization [20]. Given the enormous potential of CpG 7909 in traditional vaccine research, the adjuvant may help to address the weak immunogenicity of the YF inactivated vaccine.

In this study, we developed a YF inactivated vaccine based on Vero cell culture, the physicochemical properties of which were detected. The vaccine could avoid the adverse events of the live attenuated vaccine and also increase the YF vaccine production capacity. In addition, the effects of different adjuvants (aluminum and CpG adjuvants) on the immunogenicity of the YF inactivated vaccine were also studied. The humoral and cellular immunity results showed that the mixture of aluminum and CpG (20 μg/mL) can enhance the immunogenicity of the YF inactivated vaccine, which would help resolve the poor immunogenicity of the YF inactivated vaccine compared with those of the live attenuated ones.

## 2. Materials and Methods

### 2.1. YFV Production and Purification

The processes of YFV production and purification are similar to those described by Pato and coworkers [10]. The working virus seed of YF 17D-213 infects the Vero cells (WHO-approved cell line) and undergoes bioreactor culturing. The supernatant of the culture medium containing the virus was treated with β-propriolactone for 24 h at room temperature to inactivate virus, and then concentrated 100 times by using a molecular weight cutoff of 300 kDa membrane filter (Sartorius, Germany), followed by downstream chromatography purification. Chromatography was performed using Akta pilot, operated with the UNICORN software (https://www.cytivalifesciences.com/en/us/shop/chromatography/software/unicorn-7-p-05649) (Cytiva, Marlborough, MA, USA). Ion exchange (Q Sepharose Fast Flow) and Capto Core700 (Cytiva, USA) resin prepacked in an XK50 Column were used for capturing and polishing, respectively, and the entire purification process. The purified sample was subsequently filtered using 0.45 μm filter units (Sartorius, Goettingen, Germany).

### 2.2. Western Blot (WB)

We performed WB analysis on the obtained inactivated YF vaccine (Vero cells) original liquid. The complex conformation of proteins in the original liquid was destroyed in 4 × protein loading buffer (Invitrogen, Carlsbad, CA, USA) at 95 °C for 10 min. The protein was redistributed using 12% SDS-polyacrylamide gel electrophoresis (Solarbio, Beijing, China) and transferred to the PVDF membrane (Millpore, Billerica, MA, USA). The protein was immobilized on the surface of the solid phase and blocked with 5% skimmed milk powder (BD, New York, NY, USA) at room temperature for 1 h. Antibodies against YFV Envelope (E) protein (1:2000, GeneTex, Alton Pkwy Irvine, CA, USA) were incubated with the membrane at 4 °C for 16–24 h. The second antibody IgG labeled with HRP (1:8000, Cytiva, North Logan, UT, USA) was used to bind the first antibody and mixed with an ECL luminescent solution (Cytiva, USA). Finally, the chemiluminescence instrument (Amersham ImageQuant 800, Cytiva, USA) was used for imaging analysis.

### 2.3. Transmission Electron Microscopy (TEM)

The YFV sample was applied onto three-hundred-mesh copper grids coated with carbon, and the excess sample was blotted off. The grids were stained with 1% uranyl acetate for 1 min, followed by the blotting of excess stains, and were examined using the FEI Tecnai spirit at 120 kV.

### 2.4. Dynamic Light Scattering (DLS)

A Stunner instrument (Unchained Uncle, Norton, Tempe, AZ, USA) and Stunner client software (version 8) were used to determine the size distribution of the purified YFV.

### 2.5. Isoelectric Point (pI, or Point of Zero Charge)

The point of zero charge of YFV was measured using the Zetasizer Nano ZSTM (Malvern, Malvern, UK) [21]. The pH was adjusted with acetic acid and sodium hydroxide to prepare a series of pH gradient YFV solutions. The potentials of different pH samples were measured. The pH corresponding to the potential at 0 was the isoelectric point of the substance. The average of three measurements was calculated.

### 2.6. Vaccine Preparation

During vaccine preparation, the final concentration of inactivated YFV was 20 μg/mL, saline was used as a buffer, CpG7909 adjuvant (abbreviated as CpG) (Sangon Biotech Company, Shanghai, China) was fully dissolved in ultrapure water to 1 mg/mL, and YF/CpG was prepared by mixing YF with CpG at the formulated concentration. YF/Alum was prepared by mixing virus stock solution with aluminum hydroxide (0.6 mg/mL) (abbreviated as Alum) (Croda, Frederikssund, Danmark). In the preparation of YF/Alum/CpG, alum and CpG were slowly added to the inactivated virus, and the amount of CpG added was adjusted according to the final concentration. All of the samples were stirred at 4 °C for 2 h. In the immunogenicity test, the vaccine samples were prepared 24 h in advance and stored at 4 °C before use.

### 2.7. Determination of Vaccine Particle Size

The nano particle size analyzer (SHIMADZU, Nakagyo-ku, Japan) was used to measure the size distributions of YF/Alum and YF/Alum/CpG.

### 2.8. MicroBCA Protein Assay (Thermo Scientific)

Using a MicroBCA protein assay (Thermo Scientific, Waltham, MA, USA), a working reagent was prepared by mixing 25 parts of MA and 24 parts of MB with 1 part of MC (25:24:1, Reagent MA:MB:MC), and inactivated YF 17D vaccine was centrifuged at 7000× *g* for 5 min; the supernatant was aspirated, and the working solution was added to the supernatant and standard. The mixture was incubated at 60 °C for 1 h and cooled to room temperature. With a spectrophotometer set to 562 nm, the instrument zeroes on a cuvette filled only with a blank standard. The protein content in the semi-finished supernatant was calculated using the standard curve, and the adsorption rate = (1 − supernatant protein content/theoretical protein content) × 100%. The theoretical protein concentration is the final protein concentration used in the vaccine formulation.

### 2.9. High-Performance Liquid Chromatography (HPLC)

The quantitation of free CpG in the vaccine was determined using a Waters e2695 HPLC chromatograph system with UV detection. Empower 3 for LC systems software was used for the instrument control, data analysis, and acquisition. Separation and quantitation were achieved using a Waters X-Bridge C18 column, 5 μm, 4.6 mm × 250 mm. A mixture of solution-A: 50 mM TEAA in water, and solution-B: acetonitrile (0–5 min; 82% solution-A; 18% solution-B), was used for the elution of CpG. The flow rate was maintained at 1.0 mL/min. The column temperature was set to 20 °C. The system was equilibrated and saturated with the mobile phase for 60 min before the injection of the solutions. The standard sample was 20 μg/mL CpG aqueous solution. All of the samples were filtered using a 0.2 μm filter membrane before injection. A total of 10 μL of the solutions were injected into the duplicates. Quantification was achieved for the UV detection at 254 nm. All of the samples were quantitated according to the peak area. The adsorption rate was calculated using the following equation:

Adsorption rate (%) = (1 − (A_sample × C_standard)/(A_standard × C_real) × 100%

In the equation, A_sample and A_standard are the peak areas of CpG 7909 in the vaccine samples and standard CpG 7909, respectively. C_standard and C_real are the concentrations of the standards CpG 7909 and CpG 7909 in the vaccine samples, respectively.

### 2.10. Animals and Immunization Studies

The mice were 8–10 weeks of age (Balb/C mice, purchased from SPF Biotechnology Co., Ltd. (Beijing, China). To compare the immunogenicity of vaccine candidates with different adjuvant types and contents, 10 female mice in each group were intramuscularly injected with the vaccine candidate samples on Days 1 and 28, respectively, at a dose of 0.5 mL; YF attenuated vaccine (Beijing Institute of Biological Products Company Limited, Beijing, China) was administered with only 1 dose on Day 1, and the animals were bred in an environment free of specific pathogens (SPF). Sera were collected on Days 14, 28 (before the second dose), 35, and 49, and the binding and neutralizing antibodies were detected. On Day 35, spleen cells were collected for the cellular immunity assay.

All of the animals were kept in an environment with a temperature of 18–28 °C and a relative humidity of 40–70% and were free to drink and eat, with a light/dark cycle of 12 h. This study was conducted in a facility accredited by the Association for Evaluation and Certification of Laboratory Animal Care (AAALAC); approval was obtained from the Institutional Animal Care and Use Committee (IACUC).

### 2.11. YFV E Protein Binding Antibody Assay

The titer of YFV E protein-binding antibody in the serum samples was detected via indirect ELISA. The 96-well microtiter plates (Corning, New York, NY, USA) were pre-coated with E protein overnight at 2–8 °C and blocked with 3% BSA for 1.5 h at 37 °C. The serum was tested at a starting dilution of 1:200 with 1% BSA in PBST (PBS containing 0.05% Tween 80) and incubated at 37 °C for 1 h. After being washed five times with PBST, the serum was incubated with Amersham ECL Mouse IgG, HRP-linked whole Ab (Cytiva, North Logan, UT, USA) at 1:6000 dilution at 37 °C for 1 h. The plate was developed using TMB, following the addition of 2M H_2_SO_4_ to stop the reaction, and read at 450/630 nm with the ELISA plate reader to obtain the final data. The mean OD value of serum in the 1:100 dilution negative control group multiplied by 2.1 was used as the threshold value.

### 2.12. Plaque Reduction Neutralization Test (PRNT)

Vero cells were cultured in 199 media containing 10% FBS, with 5% CO_2_ in a 37 °C incubator. The cells were digested using 0.05% trypsin and plated to 6 × 10^5^ cells/well. After 48 h, the cells were determined to be at 80% confluence. The serum samples were treated at 56 °C for 30 min, and consecutively diluted 4-fold. An equal volume of virus dilution (17D-213, vaccines strain) was added to the serum at a titer of 250 PFU/mL and incubated at 37 °C for 60 min. The cell medium was removed, replaced with 0.4 mL virus-serum mixture, and incubated at 37 °C for 60 min. After incubation, the mixture was replaced with 3 mL of 0.75% Methyl cellulose. After 7 days, the cells were fixed with formalin (10%), stained with crystal violet (1%), and plaque counts were performed [22]. The titer end point was defined as a percentage plaque reduction of 50% (PRNT_50_).

### 2.13. FACS Assay

The cells prepared from spleens were plated on 96-well plates at ≥5 × 10^5^ cells/well in FACS solution, which consisted of PBS with 1% FBS (Gibco, Grand Island, NY, USA), and were incubated with anti-CD16/CD32 antibody (53–6.7; Biolegend, San Diego, CA, USA). For live/dead and surface staining, the cells were stained with fluorescence-labelled antibodies at 4 °C for 30 min. The live/dead antibody was Fixable Viability Dye 510 (eBioscience, San Diego, CA, USA). The surface antibodies for B cells included Pacific Blue™ anti-mouse CD45 Antibody (S18009F, Biolegend, USA), Alexa Fluor^®^ 700 anti-mouse/human CD45R/B220 Antibody (RA3-6B2, Biolegend, UAS), FITC anti-mouse/human GL7 Antigen (T and B cell Activation Marker) Antibody (GL7, Biolegend, USA), and PE anti-mouse CD95 (Fas) Antibody (SA367H8, Biolegend, USA). The surface antibodies for CD4 T cells included Alexa Fluor^®^ 700 anti-mouse CD45 Recombinant Antibody (QA17A26, Biolegend, USA), FITC anti-mouse CD90.2 (Thy-1.2) Antibody (53–2.1, Biolegend, USA), Brilliant Violet 605™ anti-mouse CD4 Antibody (GK1.5, Biolegend, USA), PerCP anti-mouse/human CD44 Antibody (IM7, Biolegend, USA), and Pacific Blue™ anti-mouse CD62L Antibody (MEL-14, Biolegend, USA). After washing with FACS solution, the cells were fixed using BD Cytofix/Cytoperm Fixation/Permeabilization Solution Kit (BD Biosciences) for 20 min at room temperature. The cells were washed twice and resuspended in FACS solution. The FACS data were obtained using a four-laser (405, 488, 561, and 638 nm) CytoFlex S (Beckman Coulter, Indianapolis, IN, USA) and analyzed using the software on the instrument.

### 2.14. ELISPOT Assay

Under sterile conditions and using an aseptic technique, the Mouse IFN-γ precoated ELISPOT kit (Dakewe Biotech Co., Ltd., Shenzhen, China) and Mouse IL-4 precoated ELISPOT kit (Dakewe Biotech Co., Ltd., China) were prepared and used according to the manufacturer’s instructions.

The ELISpot plates were activated with 200 µL serum-free medium or RPMI-1640 medium (Gibco, USA) for 5 to 10 min and dumped. Then, the cells prepared from spleens were incubated with a concentration of 2 × 10^5^ cells/well at 37 °C in a 5% CO_2_ incubator for 24 to 36 h in the presence of a stimulant (inactivated YFV), for which the concentration was 10 µg/well. PMA (Phorbol 12-myristate 13-acetate) from the kit and RPMI 1640 medium were used as positive and negative stimulants, respectively.

After incubation, the cells were removed from the plates, and the plates were washed 5 times with 200 μL of 1 × Washing buffer provided by the kit, and then incubated with 100 μL of biotinylated anti IFN-γ antibody or biotinylated anti IL-4 antibody per well for 1 h at 37 °C. After washing it 5 times with 200 μL of 1 × Washing buffer, 100 μL working concentration of streptavidin-alkaline phosphate conjugate was added per well and incubated for 1 h at 37 °C. The plates were washed 5 times with 200 μL of 1 × Washing buffer, and 100 μL of AEC was added per well. After the formation of visible spots, the reaction was terminated by rinsing the wells with distilled water. The ELISpot plate was then dried. The spots were inspected and counted with the AID EliSpot 8.0 reader system (Autoimmun Diagnostika GmbH, Strassberg, Germany). The ELISpot units were defined as spots per 2 × 10^5^ cells.

### 2.15. Statistical Analysis

Antibody titers, cytokines, and the other data were statistically analyzed using GraphPad Prism (8.0) software; the mean and standard deviation (mean ± S.D.) were calculated. Groups with normal distribution were compared via one-way ANOVA and Tukey’s test. Significance is indicated with stars: * *p* < 0.05; ** *p* < 0.01; *** *p* < 0.001; and **** *p* < 0.0001, and the non-significant groups are not shown.

## 3. Results

### 3.1. The Properties of Purified YFV

**Figure 1:**
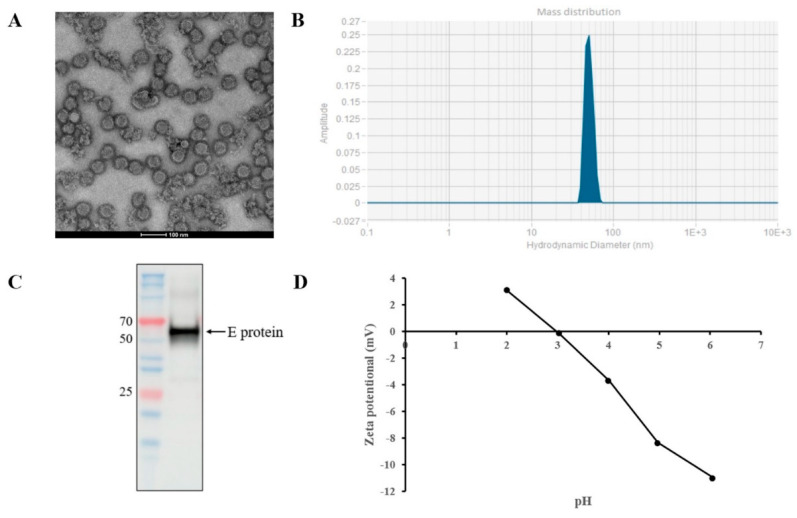
The properties of purified YFV. (**A**) Transmission electron micrographs of purified YFV sample. Scale bars, 100 nm. (**B**) Size distribution of particles in purified YFV sample measured via DLS. (**C**) The result of WB analysis of YFV E protein. Lane 1, molecular mass marker (unit, kDa); lane 2, purified YFV sample. (**D**) Isoelectric point of YFV sample.

The typical feature of YFV is the observation of spherical particles with a diameter of approximately 50 nm via regular negative staining electron microscopy [10,23]. Figure 1A shows the expected size and morphology of the purified inactivated YFV particles, confirming the successful production and purification of YFV, which can maintain the integrity of the virus particles. To confirm the size of the inactivated YFV, further analyses were carried out using DLS. The narrow size distribution indicated an average diameter of 53 nm with PDI of 0.07 (Figure 1B). The presence of the envelope protein that presents major neutralizing determinants was confirmed via Western blot [24]. The results show a specific band at 50–70 kDa (Figure 1C), which is consistent with the size of the YFV E protein (about 53 kDa). The graph of pH and potential indicates that the PI of the YFV sample is about three (Figure 1D).

### 3.2. The Properties of the Vaccine

**Figure 2:**
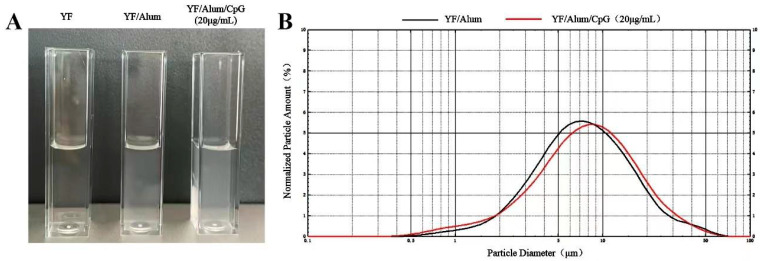
The properties of vaccine. (**A**) Appearance of YF, YF/Alum, and YF/Alum/CpG (20 μg/mL). (**B**) Size distributions of YF/Alum and YF/Alum/CpG (20 μg/mL).

YF/Alum and YF/Alum/CpG (20 μg/mL) appeared as a homogeneous suspension (Figure 2A). The particle size distribution was determined, and the results showed that the average particle sizes were 7.212 μm in the YF/Alum group and 7.380 μm in the YF/Alum/CpG (20 μg/mL) group, respectively. There was no significant increase in the particle size after adding CpG (Figure 2B).

The MicroBCA protein assay was used to determine the adsorption rate of YF in the vaccine containing the aluminum adjuvant. The results showed that the adsorption rate of YF was 100% in both YF/Alum group and YF/Alum/CpG (20 μg/mL) group (Table S1), indicating that YF could be adsorbed onto the aluminum adjuvant; this is related to both of the isoelectric points. As summarized in Table S2, the inactivated YFV with the CpG adjuvant contains many free CpGs, and the adsorption rate of CpG in the inactivated YFV formulated with both the aluminum and CpG adjuvants was 90%. The results indicate that CpG can be adsorbed with aluminum.

### 3.3. Detection of Antibodies in the YF Vaccine with Different Adjuvants

**Figure 3:**
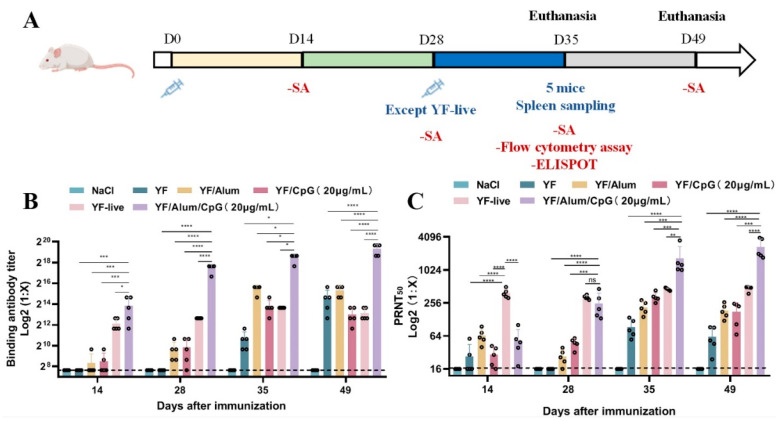
Effects of different adjuvant or vaccine types on humoral immunity in mice after vaccination. (**A**) Experimental schedule. Balb/c mice were intramuscularly injected twice on Days 0 and 28. On Days 14, 28, 35, and 49, blood was collected for serological assays (SA) (*n* = 5). On Day 35, Balb/c mice were sacrificed for enzyme-linked immunospot (ELISPOT) and flow cytometry assay (*n* = 5). (**B**) Results of binding antibody levels against YFV E protein in serum after immunization. (**C**) Results of PRNT_50_ in sera against YFV after immunization with different formulations of vaccines. Each circle represents a sample. The differences between groups were analyzed using one-way ANOVA. * *p* < 0.05; ** *p* < 0.01; *** *p* < 0.001; **** *p* < 0.0001.

We conducted a mouse study to select the best combination of the antigen and adjuvant. The Balb/c mice were injected once at intervals of 4 weeks the inactivated YF 17D vaccine with no adjuvant or adjuvants, Alum, CpG, or Alum/CpG, and once alone with YF-live on Day 1. All of the groups performed serological analysis (SA) at fixed time points (Figure 3A). Based on the YFV E protein antibody titer detection results (Figure 3B), the titer in the YF/Alum/CpG group was significantly higher than those of the other groups at all the time points, and it increased over time. The analysis of the collected animal sera with PRNT_50_ is shown in Figure 3C. The YF live vaccine induced higher antibody titers on Day 14 than the other groups achieved (*p* < 0.0001) and was stable during the follow-up. However, 28 days after the first vaccination, the PRNT_50_ increased rapidly in the YF/Alum/CpG group, with no significant difference compared with that of the YF-live group. After the second round of immunization (Day 28), the PRNT_50_ of YF/Alum/CpG group increased significantly on Days 35 and 49 compared with those of the other groups.

These results suggest that Alum/CpG is superior as a YF adjuvant to the other adjuvant combinations and live attenuated YF vaccines.

### 3.4. Antibody Responses of Different CpG Contents in YF/Alum/CpG

**Figure 4:**
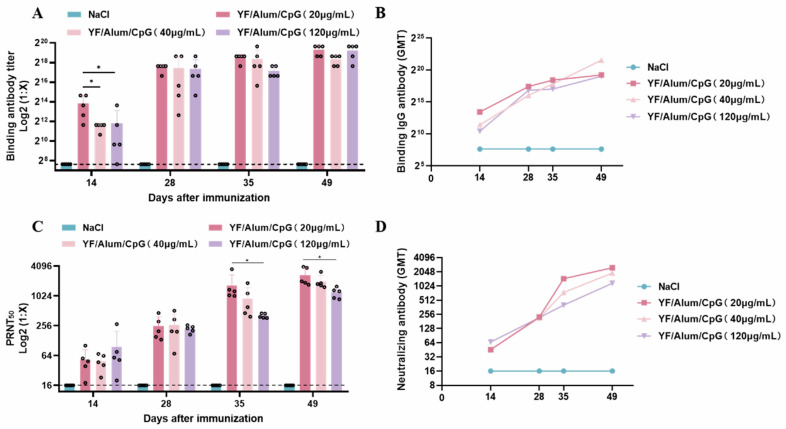
Humoral immunity in mice after immunization with different CpG doses of vaccine. (**A**) Results of binding antibody levels against YFV E protein in sera after immunization. (**B**) Determination of binding antibody GMTs. (**C**) Results of PRNT_50_ against YFV in serum after immunization. (**D**) Determination of neutralizing antibody GMTs. Each circle represents one sample. Univariate analysis of variance was used for differences between groups. * *p* < 0.05.

Three CpG doses of inactivated YFV vaccine were used to immunize the mice. Blood was collected on Days 14, 28, 35, and 49 after primary immunization, and the YFV E protein antibody titers were detected (Figure 4A,B). The results showed that on Day 14 after primary immunization, the geometric mean titer (GMT) value of the YF/Alum/CpG (20 μg/mL) group was significantly higher than those of the other groups. The YFV E protein antibody titers increased with time, and no downward trend was observed. The PRNT_50_ results showed that there was a significant difference between the YF/Alum/CpG (20 μg/mL) and YF/Alum/CpG (120 μg/mL) groups on Days 35 and 49 (*p* < 0.05) (Figure 4C). The GMT results showed that the titers of YF/Alum/CpG (20 μg/mL) increased more rapidly than those of the other groups after the second immunization. The titers of the binding and neutralizing antibodies showed that the induced titers were higher when the content of CpG was 20 μg/mL.

### 3.5. Cellular Immune Response

Next, we sought to test the induced immune responses to the candidate vaccine at the B and T cell levels. The mice were immunized with a variety of inactivated YF vaccines, which included the non-adjuvant inactivated YF vaccine, Alum inactivated YF vaccine, CpG inactivated YF vaccine, and Alum/CpG inactivated YF vaccine on Days 0 and 28, respectively. In addition, YF-live was used as a positive control to vaccinate the mice on Day 0 only. Spleen cells were obtained for cell analysis on Day 35. To investigate these targets, we performed FACS and ELISPOT assays.

The population of germinal center B cells (GCB) in the B cells was analyzed via FACS. GCB cells are closely related to the secretion of specific antibodies [25]. The above humoral immunity results suggest that Alum/CpG could significantly enhanced the antibody response of the mice and was a potent adjuvant for humoral immunity to the inactivated YF vaccine, although the GCB results did not show a clear advantage between the YF/Alum/CpG (20 μg/mL) and other experimental groups (Figure S1).

**Figure 5:**
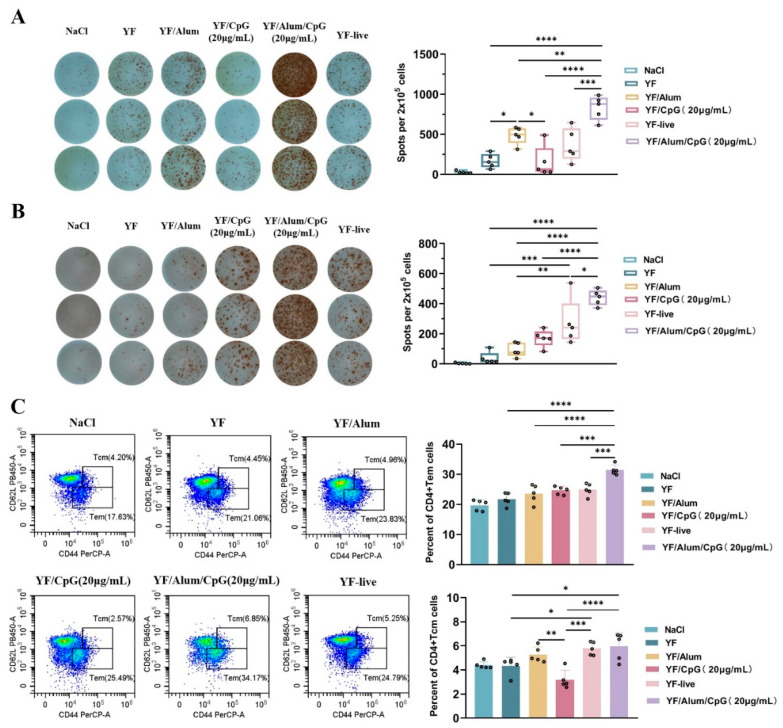
Alum/CpG is a potent adjuvant for inactivated YF vaccine given to mice measured at the cellular level. (**A**) The result of specific T cells which secreting IL-4 in spleen cells one week after two doses of immunization detected via ELISPOT assay. (Left) Representative picture of ELISPOT spots, *n* = 3. (Right) Statistical graph of specific T cells which secreting IL-4, *n* = 5. (**B**) The result of specific T cells which secreting IFN-γ in spleen cells one week after two doses of immunization detected via ELISPOT assay. (Left) Representative picture of ELISPOT spots, *n* = 3. (Right) Statistical graph of specific T cells which secreting IFN-γ, *n* = 5. (**C**) The percentage of CD4+ Tem and Tcm cells in spleen cells of mice one week after two doses of immunization. (Upper) Representative scatter plots showing CD4+ Tem and Tcm cells phenotype in splenocytes. CD4+ Tem cells were stained with fluorescent-labeled antibodies: Live + CD45+ CD90.2+ CD4+ CD44+ CD62L−; CD4+ Tcm cells were stained with fluorescent-labeled antibodies: Live + CD45+ CD90.2+ CD4+ CD44+ CD62L+. (Lower left) Statistical analysis of CD4+ Tem cells. (Lower right) Statistical analysis of CD4+ Tcm cells. Each symbol represents a mouse, and the bar represents the mean; *n* = 5 mice. Statistical analysis was performed via one-way Anova, followed by Tukey’s post-test. *, *p* < 0.05; **, *p* < 0.01; ***, *p* < 0.001; ****, *p* < 0.0001.

To explore the effects of the inactivated YF vaccine with Alum/CpG on the T cell responses, the distribution of T cell subsets were examined using ELISPOT and FACS, respectively. The T cells specifically secreting IL-4 and IFN-γ were analyzed using ELISPOT (Figure 5A,B); the CD4 memory T cell sub-cohorts were measured via FACS (Figure 5C). When immunized with Alum/CpG, the amount of specific T lymphocytes secreting IL-4 and IFN-γ was the highest, and there were significant differences compared with the group with no adjuvant, Alum, or CpG. In particular, both levels were also significantly higher than those of the YF-live, suggesting that both the Th1 and Th2 immune responses can be induced efficiently by the inactivated YF vaccine with Alum/CpG, as IFN-γ is a representative marker of Th1 immune responses, and IL-4 is a representative marker of Th2 immune responses [26].

In addition, we found that in the CD4 memory T cell subsets, the ratio of effector memory T cells (Tem) was significantly higher than those in the other groups when immunized with Alum/CpG. Although there were more central memory T cells (Tcm), there was no significant difference compared with the Alum and live attenuated YF vaccines groups. These data suggest that CD4+ Tem may be more beneficial in promoting the immunopotentiation of Alum/CpG in inactivated YF vaccines rather than CD4+ Tcm.

## 4. Discussion

There is no specific treatment for YF, and vaccination is the most effective way to prevent the disease [11]. Currently, the only available live attenuated YF vaccine is an effective formulation that is generated from chicken embryos infected with the YFV 17D strain [27]. Although the live attenuated YF vaccine has excellent immune effects, it may cause serious viscerotropic and neurotropic adverse events and anaphylaxis [14]. Inactivated vaccines based on a non-replicating cell culture would not cause these adverse events and may be used on more people, including infants under 9 months old, elderly people over 60 years old, and immunosuppressed, pregnant, and nursing people [11].

The Vero cells used are WHO-approved cell lines for vaccine production, and FBS is used during the cell growth phase, while Vero cells can be worked without FBS/FCS during viral culture. The virus titer reached its peak at 80 h and tends to be stable. In the preparation of inactivated YF virus, two detection methods were used to evaluate the inactivation effect of YF virus after inactivation with the condition. Firstly, we used a cell blind passage assay. In this experiment, inactivated YF virus was inoculated into Vero cells and observed for CPE for 7 days at which time a blind passage was performed into fresh cultures which were observed for another 7 days, blind passaged a second time and observed for 21 days, and cytopathy was not observed. Secondly, the infectivity of inactivated YF virus was measured by a plaque assay. YF virus was completely inactivated.

CpG is usually given alone or in combination with Alum as a vaccine adjuvant [28,29,30,31]. Klinman et al. demonstrated that CpG oligodeoxynucleotides (ODNs) can augment the immune response to Alhydrogel-formulated vaccines [31]. In this study, it was found that the combination of Alum/CpG significantly improved humoral and cellular immunities induced by the inactivated YF 17D vaccine. Interestingly, on Day 49, the YFV E protein antibody titers in the YF/Alum, YF/CpG, and YF-live groups began to decrease, while Alum/CpG reversed this result, indicating that the combination of Alum/CpG favored the immune persistence of the inactivated YF 17D vaccine. Although the efficacy of CpG is dose-dependent [32], this study found that CpG at a dose of 20 μg/mL had the best effect on the inactivated YF 17D vaccine, suggesting that too high a dose of CpG may disrupt the Th1/Th2 balance and affect the immune response induced by the vaccine.

Neutralizing antibodies are a major factor in the prevention of YFV infections, and live attenuated vaccines always induce higher levels of neutralizing antibodies than inactivated vaccines do, which also presents new challenges for the preparation of inactivated vaccines. In this study, a YF inactivated vaccine was prepared using different adjuvants with the aim of selecting adjuvants that can induce higher levels of YF antibody titers. We used a PRNT assay to detect the neutralizing antibody titers; this method is considered to be the gold standard for evaluating the presence of neutralizing antibodies against YFV [22]. It is worth mentioning that the live attenuated vaccine group was immunized with 1 dose, and all of the other groups were immunized with two doses on Days 0 and 28. The results showed that the attenuated vaccine had an obvious advantage in inducing antibody production, and it could induce a higher antibody level within 14 days. Surprisingly, the antibody levels in the YF/Alum/CpG group were comparable to those in the attenuated vaccine-receiving group after 28 days and significantly higher than those in the Alum or CpG group. We hypothesize that this may be related to the formation of a reservoir of Alum/CpG and the enhancement of cellular immunity. It should be noted that on Days 35 and 49, we observed significantly higher antibody levels in the YF/Alum/CpG group than those in the live attenuated vaccine-receiving group, which may be explained by the fact that two doses of immunization were performed. In addition, two doses of D 0/28 significantly increased the antibody level in the YF/alum/CpG group.

The mechanisms by which the YF vaccine induces immune protection have not been well investigated. In addition to the role of neutralizing antibodies, which has been generally accepted [33], the role of T cells and cytokines has also been widely studied in recent years. Neves PC et al. demonstrated in an Indian rhesus macaque model that vaccination with YF-17D induced IFN-γ production early after vaccination (on Days 5–7) before the development of classical antigen-specific CD8 and CD4 T cell responses [34]. Santos et al. found that high levels of lymphocytes were induced, which produce IFN-γ and IL-4 after inoculation with YF-17D, indicating that the Th1 and Th2 cytokines play important roles in the immune response to the YF vaccine [35]. In addition, it has been reported that the memory phenotype of CD8+ T cells after YF-17D vaccination is mainly manifested as effector memory T cells, contributing to the effectiveness of the YF vaccine [36].

The abovementioned research is consistent with our experimental results, even though the animals we used in our study were mice, which are different from monkeys and humans. For example, we found the numbers of lymphocytes producing IFN-γ and IL-4 increased via ELISpot after immunization with the live attenuated YF vaccine. Through memory phenotype analysis, we detected that the number of CD4+ T cells, not the CD8+ T cells, and the CD4+ Tem cell phenotypes increased significantly too. It is worth noting that when we immunized the mice with the inactivated YF vaccine containing the Alum/CpG adjuvant, the number of lymphocytes producing the IFN-γ and IL-4 and CD4+ Tem cell phenotypes all significantly increased compared to that of the live attenuated vaccine. This occurred even if two doses of the inactivated vaccine were given, or one dose of the attenuated vaccine. These results suggest that the Alum/CpG inactivated YF vaccine can not only induce higher levels of neutralizing antibodies, but also further enhance the cellular immunity effect compared with those of the Alum inactivated YF vaccine, CpG inactivated YF vaccine, or live attenuated YF vaccine.

In our study, we focused on exploring the use of Alum/CpG to enhance the humoral and cellular immunities of the YF inactivated vaccine, but further investigations are needed to understand the long-term immune effects and challenge studies.

## 5. Conclusions

In conclusion, we prepared an inactivated YF vaccine and used Alum/CpG as an adjuvant. The effects of YF/Alum/CpG on humoral and cellular immunity were evaluated using a mouse model. The antibody level at day 28 after one dose was similar to that of the live attenuated vaccine. At the same time, Alum/CpG significantly increased the levels of IFN-γ and IL-4 cytokines. These results showed that it is possible to provide an effective inactivated YF vaccine with the potential to solve the shortage of live attenuated vaccine.

## Data Availability

Data are contained within the article and Supplementary Materials.

## Supplementary Materials

The following supporting information can be downloaded at: https://www.mdpi.com/article/10.3390/vaccines11121744/s1, Table S1. YF adsorption rate in aluminum-containing vaccine samples; Table S2. CpG adsorption rate in different samples; Figure S1 The percentage of GCB cells in spleen cells of mice one week after two doses of immunization. (a) Representative scatter plots showing GCB cell phenotype in splenocytes. GCB cells were stained with fluorescent labeled antibodies: Live + CD45+ B220+ GL7+ CD95+. (b) Statistical analysis of GCB cells. Each symbol represents a mouse and the bar represents the mean, *n* = 5 mice. Statistical analysis was performed by One-way anova, followed by Tukey’s post-test. *, *p* < 0.05.

## References

[1] Tuells J., Henao-Martínez A.F., Franco-Paredes C. (2022). Yellow Fever: A Perennial Threat. Arch. Med. Res..

[2] Gianchecchi E., Cianchi V., Torelli A., Montomoli E. (2022). Yellow Fever: Origin, Epidemiology, Preventive Strategies and Future Prospects. Vaccines.

[3] Chala B., Hamde F. (2021). Emerging and Re-emerging Vector-Borne Infectious Diseases and the Challenges for Control: A Review. Front. Public Health.

[4] Heinz S., Kolimenakis A., Horstick O., Yakob L., Michaelakis A., Lowery Wilson M. (2021). Systematic review: Yellow fever control through environmental management mechanisms. Trop. Med. Int. Health.

[5] Crunkhorn S. (2023). Monoclonal antibody treats yellow fever. Nat. Rev. Drug Discov..

[6] Gaythorpe K.A., Hamlet A., Jean K., Garkauskas Ramos D., Cibrelus L., Garske T., Ferguson N. (2021). The global burden of yellow fever. eLife.

[7] Diagne M.M., Ndione M.H.D., Gaye A., Barry M.A., Diallo D., Diallo A., Mwakibete L.L., Diop M., Ndiaye E.H., Ahyong V. (2021). Yellow Fever Outbreak in Eastern Senegal, 2020–2021. Viruses.

[8] Hansen C.A., Barrett A.D. (2021). The Present and Future of Yellow Fever Vaccines. Pharmaceuticals.

[9] De Andrade Gandolfi F., Estofolete C.F., Wakai M.C., Negri A.F., Barcelos M.D., Vasilakis N., Nogueira M.L. (2023). Yellow Fever Vaccine-Related Neurotropic Disease in Brazil following Immunization with 17DD. Vaccines.

[10] Pato T.P., Souza M.C., Mattos D.A., Caride E., Ferreira D.F., Gaspar L.P., Freire M.S., Castilho L.R. (2019). Purification of yellow fever virus produced in Vero cells for inactivated vaccine manufacture. Vaccine.

[11] Monath T.P., Lee C.K., Julander J.G., Brown A., Beasley D.W., Watts D.M., Hayman E., Guertin P., Makowiecki J., Crowell J. (2010). Inactivated yellow fever 17D vaccine: Development and nonclinical safety, immunogenicity and protective activity. Vaccine.

[12] Reyes C., Patarroyo M.A. (2023). Adjuvants approved for human use: What do we know and what do we need to know for designing good adjuvants?. Eur. J. Pharmacol..

[13] Laera D., HogenEsch H., O’Hagan D.T. (2023). Aluminum Adjuvants–‘Back to the Future’. Pharmaceutics.

[14] Monath T.P., Fowler E., Johnson C.T., Balser J., Morin M.J., Sisti M., Trent D.W. (2011). An inactivated cell-culture vaccine against yellow fever. N. Engl. J. Med..

[15] Pereira R.C., Silva A.N., Souza M.C.O., Silva M.V., Neves P.P., Silva A.A., Matos D.D., Herrera M.A., Yamamura A.M., Freire M.S. (2015). An inactivated yellow fever 17DD vaccine cultivated in Vero cell cultures. Vaccine.

[16] Yang J.X., Tseng J.C., Yu G.Y., Luo Y., Huang C.Y.F., Hong Y.R., Chuang T.H. (2022). Recent Advances in the Development of Toll-like Receptor Agonist-Based Vaccine Adjuvants for Infectious Diseases. Pharmaceutics.

[17] Kayraklioglu N., Horuluoglu B., Klinman D.M. (2021). CpG oligonucleotides as vaccine adjuvants. Methods in Molecular Biology.

[18] Yang J., Li B., Yang D., Wu J., Yang A., Wang W., Lin F., Wan X., Li Y., Chen Z. (2021). The immunogenicity of Alum+CpG adjuvant SARS-CoV-2 inactivated vaccine in mice. Vaccine.

[19] Cooper C.L., Angel J.B., Seguin I., Davis H.L., Cameron D.W. (2008). CPG 7909 adjuvant plus hepatitis B virus vaccination in HIV-infected adults achieves long-term seroprotection for up to 5 years. Clin. Infect. Dis..

[20] Rynkiewicz D., Rathkopf M., Sim I., Waytes A.T., Hopkins R.J., Giri L., DeMuria D., Ransom J., Quinn J., Nabors G.S. (2011). Marked enhancement of the immune response to BioThrax^®^ (Anthrax Vaccine Adsorbed) by the TLR9 agonist CPG 7909 in healthy volunteers. Vaccine.

[21] Huang M., Wang W. (2014). Factors affecting alum-protein interactions. Int. J. Pharm..

[22] Simões M., Camacho L.A.B., Yamamura A.M., Miranda E.H., Cajaraville A.C.R., da Silva Freire M. (2012). Evaluation of accuracy and reliability of the plaque reduction neutralization test (micro-PRNT) in detection of yellow fever virus antibodies. Biologicals.

[23] Lima T.M., Souza M.O., Castilho L.R. (2019). Purification of flavivirus VLPs by a two-step chomatographic process. Vaccine.

[24] Crill W.D., Chang G.J.J. (2004). Localization and characterization of flavivirus envelope glycoprotein cross-reactive epitopes. J. Virol..

[25] Liu B., Lin Y., Yan J., Yao J., Liu D., Ma W., Wang J., Liu W., Wang C., Zhang L. (2021). Author Correction: Affinity-coupled CCL22 promotes positive selection in germinal centres. Nature.

[26] Mousset C.M., Hobo W., Woestenenk R., Preijers F., Dolstra H., van der Waart A.B. (2019). Comprehensive Phenotyping of T Cells Using Flow Cytometry. Cytom. Part A.

[27] Ivanov A.P., Klebleeva T.D., Rogova Y.V., Ivanova O.G.E. (2020). Development of inactivated cultural yellow fever vaccine. Probl. Virol..

[28] Campbell J.D. (2017). Development of the CpG adjuvant 1018: A case study. Methods in Molecular Biology.

[29] Biryukov S., Dankmeyer J.L., Shamsuddin Z., Velez I., Rill N.O., Rosario-Acevedo R., Klimko C.P., Shoe J.L., Hunter M., Ward M.D. (2021). Impact of Toll-Like Receptor-Specific Agonists on the Host Immune Response to the Yersinia pestis Plague rF1V Vaccine. Front. Immunol..

[30] Liang J.G., Su D., Song T.Z., Zeng Y., Huang W., Wu J., Xu R., Luo P., Yang X., Zhang X. (2021). S-Trimer, a COVID-19 subunit vaccine candidate, induces protective immunity in nonhuman primates. Nat. Commun..

[31] Higgins D., Marshall J.D., Traquina P., Van Nest G., Livingston B.D. (2007). Immunostimulatory DNA as a vaccine adjuvant. Expert Rev. Vaccines.

[32] Kim Y.H., Lee S.H., Yoo Y.C., Lee J., Park J.H., Park S.R. (2012). Kinetic Analysis of CpG-Induced Mouse B Cell Growth and Ig Production. Immune Netw..

[33] Poland J.D., Calisher C.H., Monath T.P., Downs W.G., Murphy K. (1981). Persistence of neutralizing antibody 30–35 years after immunization with 17D yellow fever vaccine. Bull. World Health Organ..

[34] Neves P.C., Rudersdorf R.A., Galler R., Bonaldo M.C., de Santana M.G.V., Mudd P.A., Martins M.A., Rakasz E.G., Wilson N.A., Watkins D.I. (2010). CD8+ gamma-delta TCR+ and CD4+ T cells produce IFN-γ at 5–7 days after yellow fever vaccination in Indian rhesus macaques, before the induction of classical antigen-specific T cell responses. Vaccine.

[35] Santos A.P., Matos D.C.S., Bertho A.L., Mendonça S.C.F., Marcovistz R. (2008). Detection of Th1/Th2 cytokine signatures in yellow fever 17DD first-time vaccinees through ELISpot assay. Cytokine.

[36] Co M.D.T., Kilpatrick E.D., Rothman A.L. (2009). Dynamics of the CD8 T-cell response following yellow fever virus 17D immunization. Immunology.

