# Supplementary material for: Enhancing Inactivated Yellow Fever 17D Vaccine-Induced Immune Responses in Balb/C Mice Using Alum/CpG
Source: Vaccines (Basel). 2023 Nov 22;11(12):1744. doi: 10.3390/vaccines11121744 (PMC10747526; doi:10.3390/vaccines11121744)
Supplement: Supplementary file 1 [file vaccines-11-01744-s001.zip › vaccines-2678296-supplementary.pdf]

Supporting Information

For

Enhancing Inactivated Yellow Fever 17D Vaccine−Induced Immune Responses in Balb/C Mice Using Alum/CpG

Yadan Zhang[1], Rong Yang[1], Guangying Yuan[1], Weidong Li[1], Zihao Cui[1], Zhuangzhuang Xiao[1], Xiaofei Dong[1], Hongqiang Yang[1], Xiaojuan Liu[1], Le Zhang[1], Yirong Hou[1], Manyu Liu[1], Sushi Liu[1], Yu Hao[1], Yuntao Zhang[1*], Xiaotong Zheng[1, *]

[1]Beijing Institute of Biological Products Company Limited, Beijing, China

*Correspondence: zhangyuntao@sinopharm.com (Y.Z.), zhengxiaotong@sinopharm.com(Z.X）

Supplementary Table S1. YF adsorption rate in aluminum-containing vaccine samples

Samples	Concentration of Alum	YF Adsorption rate
YF/Alum	0.6mg/mL	100%
YF/Alum/CpG（20μg/mL）	0.6mg/mL	100%

Supplementary Table S2. CpG adsorption rate in different samples

Samples	Concentration of CpG	Retention time	The average of peak areas	Concentration of free CpG	Adsorption rate
Standard CpG 7909	20 μg/mL	3.2 min	250645.9	——	——
YF/CpG	20 μg/mL	3.2 min	221397.5	17.7 μg/mL	11.5%
YF/Alum/CpG	20 μg/mL	3.2 min	24256.5	1.9 μg/mL	90.5%

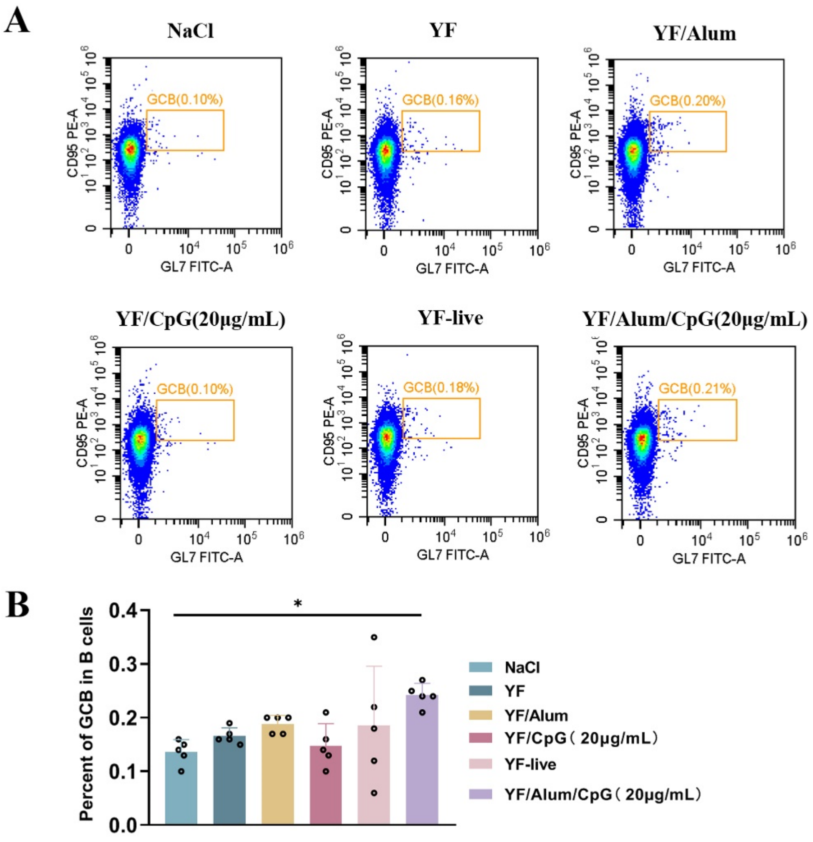

Supplementary Figure S1. The percentage of GCB cells in spleen cells of mice one week after two doses of immunization. (**a**) Representative scatter plots showing GCB

cell phenotype in splenocytes. GCB cells were stained with fluorescent labeled antibodies: Live+CD45+B220+GL7+CD95+. (**b**) Statistical analysis of GCB cells. Each symbol represents a mouse and the bar represents the mean, n = 5 mice. Statistical analysis was performed by One-way Anova, followed by Tukey's post-test. *, $p < 0.05$.
